# Daily application of low magnitude mechanical stimulus inhibits the growth of MDA-MB-231 breast cancer cells in vitro

**DOI:** 10.1186/s12935-014-0102-z

**Published:** 2014-10-14

**Authors:** Melis Olcum, Engin Ozcivici

**Affiliations:** Program in Biotechnology and Bioengineering, Izmir Institute of Technology, Urla, Izmir Turkey; Department of Mechanical Engineering, Izmir Institute of Technology, Urla, Izmir Turkey

**Keywords:** Mechanical loading, MDA-MB-231, Physical activity, Breast cancer

## Abstract

**Introduction:**

Mechanical loads can regulate cell proliferation and differentiation at various stages of development and homeostasis. However, the extension of this regulatory effect of mechanical loads on cancer cells is largely unknown. Increased physical compliance is one of the key features of cancer cells, which may hamper the transmission of mechanical loads to these cells within tumor microenvironment. Here we tested whether brief daily application of an external low magnitude mechanical stimulus (LMMS), would impede the growth of MDA-MB-231 aggressive type breast cancer cells *in vitro* for 3 wks of growth.

**Methods:**

The signal was applied in oscillatory form at 90 Hz and 0.15 g, a regimen that would induce mechanical loads on MDA-MB-231 cells via inertial properties of cells rather than matrix deformations. Experimental cells were exposed to LMMS 15 min/day, 5 days/week in ambient conditions while control cells were sham loaded. Cell proliferation, viability, cycle, apoptosis, morphology and migration were tested via Trypan Blue dye exclusion, MTT, PI, Annexin V, Calcein-AM and phalloidin stains and scratch wound assays.

**Results:**

Compared to sham controls, daily application of LMMS reduced the number and viability of cancerous MDA-MB-231 cells significantly after first week in the culture, while non-cancerous MCF10A cells were found to be unaffected. Flow cytomety analyses suggested that the observed decrease for the cancer cells in the LMMS group was due to a cell cycle arrest rather than apoptosis. LMMS further reduced cancer cell circularity and increased cytoskeletal actin in MDA-MB-231 cells.

**Conclusion:**

Combined, results suggest that direct application of mechanical loads negatively regulate the proliferation of aggressive type cancer cells. If confirmed, this non-invasive approach may be integrated to the efforts for the prevention and/or treatment of cancer.

## Introduction

Cancer, one of the leading causes of death worldwide, is characterized by the uncontrolled growth of cells that eventually leads to disruption of tissue organization and function [1]. Since no universal treatment is available for cancer other than remedies that kill rapidly dividing cells at the expense of life quality [2–5], prevention strategies are often emphasized for the practice of healthy lifestyle choices such as following a healthy diet, minimizing sun exposure and cessation of substance addictions [6]. One of those healthy choices is to avoid a sedentary lifestyle, and indeed a negative correlation exists between the levels of physical activity and rate of incidence for cancer [7–11]. Increased physical activity is associated with reduced incidence rates of colon, breast, prostate, endometrial and lung cancers [7,8,12,13]. Suggested mechanisms for reduced cancer incidence mainly focus on the global effects of exercise on body fat mass, hormonal or immune status [12], but recently interaction of mechanical loads with cancer cells received further attention [14–16].

Mechanical loads are omnipresent in all tissues and they act as an important modulator of cellular machinery during an organism’s development and homeostasis [17–20]. Cellular decisions such as growth, migration and differentiation are dependent on mechanical loads, and the absence of these loads induces catabolism and malformation in many tissue types. Taking advantage of the regulatory and anabolic potential of mechanical stimuli, daily physical exercise is protective against diseases that are detrimental to tissue integrity [21–23]. Though cellular response to mechanical loads in healthy and diseased tissue types were addressed frequently in the literature [24], how cancerous cells respond to mechanical loads and whether they are exempt from the regulatory effects of mechanical loads is largely unknown.

Many cancer types present themselves with a tumor formation, a structure that is significantly stiffer compared to the surrounding healthy tissue [25]. The stiffness of tumor tissue is governed by the aberrant extracellular matrix deposited by the cancer cells. In contrast, cancer cells that lie within the tumor tissue are more compliant compared to healthy cells as evidenced by single cell mechanical manipulation techniques [26]. Cancer cells can readily utilize their “more compliant state” for an increased efficacy for invasion and migration to distant sites [27]. Other than a small fraction (<6%) of cases that are formed as mucinous tumors [28] breast cancer is no exception to stiff extracellular matrix and compliant cell composition [29]. From a mechanical perspective, the composition of an extracellular matrix that is stiffer, and cells that are softer than a healthy tissue suggests that breast cancer cells may also be protected from mechanical loads in a manner that is similar to the event of “stress shielding” seen in orthopedic biomaterial applications [30,31]. In a composite structure stiffer elements absorb larger loads compared to compliant elements. Even though cancer cells are known to be responsive to mechanical cues [32,33], if they are protected from global loads within stiffer tumors, then potential regulatory effects of these loads on cancer cells may not be able to potentiate in the first place.

Alternative to external loads that are prescribed to tissues and shared between extracellular matrix and cells, accelerations can be used to generate mechanical loads on the cells based on Newton’s 2^nd^ law of motion. In such a system, accelerations would prescribe mechanical loads on every element of the system based on the mass of individual elements. These repeated oscillatory loads need not to be large in magnitude, as healthy tissues and cells can sense and respond to these loads that are at least two orders of magnitude smaller compared to mechanical loads that are associated with regular weight bearing [34–36]. Here we tested whether direct application of low magnitude mechanical stimuli (LMMS) would be detrimental to the proliferation and invasiveness of aggressive type human breast cancer cells.

## Materials and methods

All experiments were conducted in compliance with ethical board of Izmir Institute of Technology. MDA-MB-231 (American Type Culture Collection, VA, USA) aggressive type breast cancer cells were used throughout the study. Briefly, cells were cultured using DMEM with high glucose (Thermo Scientific HyClone, UT, USA) supplemented with 1% Pen/Strep (Biological Industries, Israel) and 10% FBS (Biological Industries, Israel). MCF10A (American Type Culture Collection, VA, USA) human breast epithelial cells were used as non-cancerous epithelial controls. MCF10A cells were cultured in DMEM:F12 medium (Sigma, MO, USA) supplemented with 20 ng/ml EGF (Sigma, MO, USA), 0.5 ug/ml hydrocortisone (Sigma, MO, USA), 100ng/ml choleratoxin (Sigma, MO, USA), 10 ug/ml insulin (Sigma, MO, USA), 1% Pen/Strep (Biological Industries, Israel), 5% donor equine serum and 2 mM L-glutamine. Cells were kept in 37°C and 5% CO_2_ except vibratory loading protocol, during which they were exposed to the ambient conditions. For all experiments cells were cultured in 24 well plates (Corning, NY, USA) with a 640 cells/mm^2^ density to prevent overpopulation during 3 wks and culture medium was changed every two days. For all experiments, day of plating was considered as day (-2) and cultures were maintained for a range of days, including D1, D3, D5, D9, D12 and D19 (Table 1). At the designated time points, experiments were terminated for further analysis.

**Table 1:** **Experimental design calendar for the application of sham signal or low magnitude mechanical stimulus (LMMS) to MDA-MB-231 breast cancer cells**

**D(-1)**	**D0**	**D1**	**D2**	**D3**	**D4**	**D5**
○	○	X,^†^	X	X,^†^	X	X,^†^
**D6**	**D7**	**D8**	**D9**	**D10**	**D11**	**D12**
○	○	X	X,^†^	X	X	X,^†^
**D13**	**D14**	**D15**	**D16**	**D17**	**D18**	**D19**
○	○	X	X	X	X	X,^†^

X, LMMS/Sham; ○, Rest; ^†^, Evaluation.

After plating cells on D(-2), LMMS or sham signal was applied starting from D1 until D19, 15 min/day, 5 days/week. Cultures were terminated on selected days for the evaluation of cellular indices.

Experimental cells were exposed to a daily regimen of mechanical vibration at 90 Hz and 0.15 g (1 g = Earth’s gravitational pull), for 15 min/day, 5 days/week in ambient conditions, during which control plates were subjected to sham loading. Mechanical signal was provided by a custom-made platform in vertical direction [37], and the mechanical signal quality was continuously controlled with real-time accelerometer (K-Beam, Kistler, Amhers, NY, USA), measurements monitored by Labview 2010 Signal Express (National Instruments, Austin, TX, USA) software.

Number of cells for experimental and control groups were quantified using trypan blue exclusion method, where cells were diluted with 0.4% trypan blue dye (Gibco, Invitrogen, NY, USA) dye in 1:1 ratio and counted with a Neubauer hemocytometer. Cell viability was analyzed via MTT assay, in which cells were incubated with 0.5 mg/ml MTT (Amresco LLC, OH, USA) for 4 hours. After the incubation tetrazolium salts were dissolved in DMSO and colorimetric measurements was done at 570 nm with a background subtraction at 650 nm. Cellular morphology and actin ultrastructure was visualized using phalloidin (Invitrogen, USA) staining followed by analysis of individual cells using Image J software.

Experimental and control groups were subjected to cell cycle analysis based on the DNA content of the cells via propidium iodide (PI) staining. Briefly, cells were collected in 1x cold PBS solution and then fixed with EtOH. After overnight incubation at -20°C, cells were permeabilized with 0.1% Triton x-100 in PBS and treated with RNase A. Finally, cells were incubated with PI and analyzed with FACS Canto (BD Biosciences, CA, USA) with low flow rate. Based on binomial distribution of PI signal gating and doublet distinction was done according to the area of the signal peaks with a cell cycle analysis software (Modfit LT, Verity Software, USA). Fractions of apoptotic, live and dead cells were quantified using Annexin V – PI (BD Pharmingen, NJ, USA) staining based on the specifications instructed by the provider. Briefly, collected cells were washed twice with PBS then suspended in binding buffer and stained with Annexin V (FITC) and PI dyes. Cells were analyzed with FACS Canto where single stain and unstained cells were used to set event gates. Calcein-AM cell permeant dye (Life Technologies, Oregon, USA) was used to stain MDA-MB-231 cell at days D5, D12 and D19, with 30 min incubation in the dark, followed by cell detachment and flow cytometry measurements.

Cellular morphology and actin ultrastructure was documented with phalloidin (Alexa Fluor 488, Invitrogen, USA) staining after cellular fixation (4% paraformaldehyde) and membrane permeabilization (0.1% TritonX in PBS). Images were acquired by fluorescence microscopy (CKX71, Olympus, Japan) and processed with an image processing software (Image J, USA). Scratch closure rates for both groups were quantified using an artificial scratch mark made with the tip of a 200 μl pipette. Scratches were visualized immediately and after 24 hrs of incubation for both groups using a microscope (CKX41, Olympus, Japan) with image processing software (DP2-BSW, Olympus, Tokyo, Japan). The gap between cells was measured from 3 different regions on a single scratch, repeated 10 times within sample. The percent change of average gap length between 0 and 24 hrs was reported as an indicator of gap closure. Mechanical signal was not applied to cells during this 24 hrs period.

All results were reported as mean (±standard deviation). Groups were compared using Student’s *t* test with unequal variance where statistical significance was set at 5%. For all experiments described above, a minimum of 3 replicates were used from both groups, except cell detachment and calcein stains.

## Results

**Figure 1:**
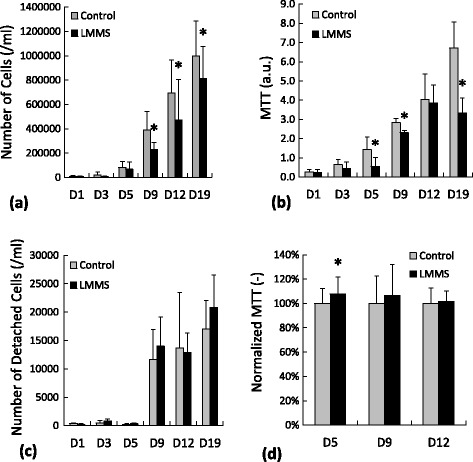
**Cell proliferation measurements of MDA-MB-231 cells for control and LMMS groups assessed with a)** Trypan Blue dye exclusion method **b)** MTT assay. **c)** Number of MDA-MB-231 cells that were detached from the plastic counted by Trypan Blue stain. **d)** Cell proliferation of MCF10A cells assessed with MTT assay. (*: p < 0.05 between LMMS and sham controls).

**Figure 2:**
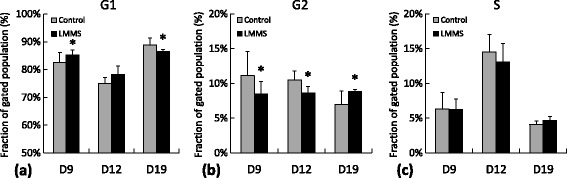
**MDA-MB-231 cell cycle assessment showing cell fractions for control and LMMS groups that were in a)** G1 phase, **b)** G2 phase and **c)** S phase, represented based on experimental days 9, 12 and 19. (*: p < 0.05 between LMMS and controls).

During 19 days of culture a steady increase was observed in sham control MDA-MB-231 cells as measured with Trypan Blue cell counts. At the end of 19 days, on average number of cells increased 108-fold compared to baseline controls (Figure 1a). Number of low magnitude mechanical stimulation (LMMS) treated cells also showed a steady increase during experimental period with an average increase of 92-fold compared to baseline controls. LMMS group had 41%, 32% and 18% (all p < 0.05) less number of MDA-MB-231 cells at D9, D12 and D19 compared to controls. Similar to Trypan blue readings, cell viability signals of MDA-MB-231 as documented with MTT assays for sham control cells showed a steady increase during the experimental protocol (Figure 1b). At D19 MTT signal showed 25-fold increase compared to baseline controls. Compared to sham controls, LMMS treated cells showed 62%, 18% and 50% (all p < 0.05) less MTT signal during D5, D9 and D19, respectively. To test if the reduced number of cells in LMMS groups observed because of cellular detachment from culture plate, cells in collected media was counted for both groups using Trypan blue stain (Figure 1c). No significant differences (all p > 0.1) were detected for number of detached cells in experimental days (Figure 2c). In spite of observed reduction in cell numbers for breast epithelial cancer cells (MDA-MB-231), similar loading with LMMS affected non-cancerous breast epithelial cells (MCF10A) differently (Figure 1d). At the D5, LMMS group had 8% more MTT activity (p < 0.01) compared to controls. At D12 and D19, MTT activity of MCF10A cells in LMMS group had a non-significant 6% and 1% (p = 0.20 and 0.55, respectively) difference compared to sham controls.

In an effort to explain the reduction of cell numbers and viability in LMMS treated cultures, cell cycle analysis was performed using PI staining. Unfortunately, cultures from both sham control and LMMS groups at D1, D3 and D5 lacked enough number of cells for analysis therefore cultures from D9, D12 and D19 were reported here. At D9, LMMS group had 3% (0.03) more fraction of cells in G1 phase while 24% (p = 0.03) less fraction of cells in G2 phase compared to sham controls (Figure 2a and b). At D12, LMMS group had 18% (p = 0.02) less fraction of cells in G2 phase. In contrast to previous time points, LMMS treated cells at D19 had 3% (p = 0.05) less fraction of cells at G1, while 27% (p = 0.05) more fraction cells in G2 compared to controls. No significant differences were observed in the fraction of cells that are in S phase between LMMS and controls for the duration of experiment (Figure 2c).

**Figure 3:**
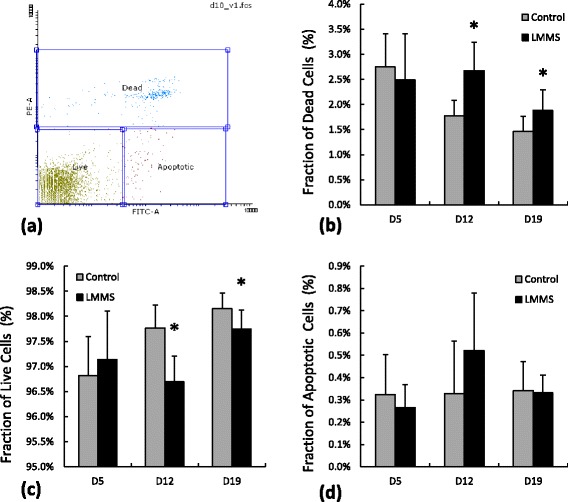
**MDA-MB-231 apoptosis/necrosis assessment with Annexin V – PI stains of control and LMMS groups for different experimental days. a)** Representative gates applied for all samples determining dead, live and apoptotic cells. Fractions of **b)** dead cells (PI+), **c)** live cells (No stain) and **d)** apoptotic cells (Annexin V+, PI-) (*: p < 0.05 between LMMS and controls).

Control and LMMS cells were stained with Annexin V – PI documented for the apoptotic status of experimental cells (Figure 3a). No difference (all p > 0.15) in the fraction of apoptotic cells were observed between control and LMMS groups (Figure 3d). Fraction of dead cells in LMMS group was 51% (p < 0.01) and 28% (p = 0.05) larger at D12 and D19 compared to controls (Figure 3b). At D12, fraction of live cells had small but a significant reduction (1.1%, p < 0.01) compared to control cells (Figure 3c). Furthermore, fraction of live cells at D12 showed a similar reduction (3%, p = 0.02) as determined by Calcein-AM staining (Table 2).

**Table 2:** **Calcein-AM staining of MDA-MB-231 breast cancer cells at different experimental days**

	**D5**	**D12**	**D19**
**Control**	93.3 ± 1.4	92.1 ± 1.0	92.8 ± 1.2
**LMMS**	94.7 ± 1.4	89.5 ± 1.2*	93.5 ± 0.6

(*: p < 0.05).

**Figure 4:**
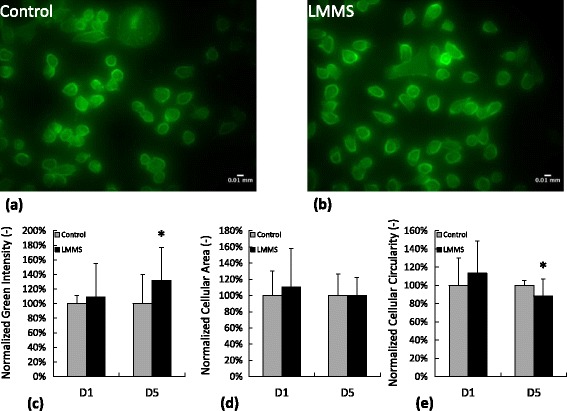
**Phalloidin staining and morphology of MDA-MB-231 cells for control and LMMS groups for experimental days 1 and 5.** Representative micrographs from **a)** control and **b)** LMMS group. Individual cells were analyzed for **c)** mean green intensity, **d)** cellular area and **e)** cellular circularity. (*: p < 0.05 between LMMS and controls).

**Figure 5:**
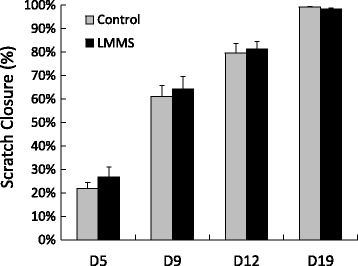
**Scratch closure rates (%) of MDA-MB-231 cells for control and LMMS groups for the duration of 24 hours represented based on experimental days 5, 9, 12 and 19.**

The effect of LMMS on the morphology and ultrastructure of MDA-MB-231 cells were determined by fluorescence microscopy (Figure 4a and b). At D1 no significant difference was observed in individual cellular area, cellular circularity and actin content (Figure 4c-e). However at the end of first week cancer cells that received daily LMMS had 32% (p = 0.02) stronger fluoresce signal with 12% (p = 0.04) smaller circularity compared to control cells. Migratory potential of MDA-MB-231 increased with the confluence during the experimental protocol as evidenced by 24 hrs scratch closure rates. However, daily application of LMMS did not affect scratch closure significantly for the time points analyzed (Figure 5).

## Discussion

The ability of low magnitude mechanical signals (LMMS) to interfere with the growth, viability, cell cycle, apoptosis and migration potential of MDA-MB-231 aggressive type breast cancer cell was investigated *in vitro* for 3wks duration. MDA-MB-231 cells showed a steady proliferation rate during the extension of the study. Daily application of LMMS reduced number of breast cancer cells at different time points, but did not affect non-cancerous controls. According to the cell cycle analysis, LMMS reduced the fraction of cancer cells that are cycling and arrested them in either G1 or G2 phase. However, LMMS neither interfered with the migratory potential nor induced apoptosis in MDA-MB-231 cells. Reported results indicate that the application of LMMS to breast cancer cells may suppress the uncontrolled growth, emphasizing a potential therapeutic benefit against the disease.

Implementation of LMMS for 15 min/day on MDA-MB-231 cells decreased number of cycling cells in short-term. Colon cancer cells show a similar cell cycle arrest at G1 when exposed to mechanical loads in shear form [38], perhaps signifying a global pattern for the mechanical regulation on cancer cells. Similarly, MCF-7 breast cancer cells cycle more and show increased proliferation during space-flight [39], a condition that induce constant unloading to cells [40]. Further, constant weightlessness on thyroid carcinoma cells is associated with events that increase extracellular matrix formation, metastatic spread with the inhibition of apoptosis [41]. Combined, these data emphasize that future studies should identify the mechanical regulation on the molecular mechanisms of cancer cell cycle and whether brief exposure to daily physical stimulus can impede the growth of other tissue types of cancer.

The stimulus that was investigated here was largely tested *in vivo* for the anabolic effects in skeletal tissue during and after mechanical unloading [42], for suppression of adipogenesis in fat pads [43], liver [44] by dietary induced obesity and for normalization of hematological function in bone marrow hampered by obesity [45]. None of those studies reported any adverse effects of LMMS on the tissues studied within. Recently, Pagnotti et al used a similar daily loading regimen on a mouse model of spontaneous granulosa cell ovarian cancer and showed a benefit to skeletal health without compromising the longevity of the organism [46]. These results are important in describing that low magnitude mechanical stimulus is advantageous to tissues that are threatened with several disease states without benefiting the progression of cancer. Even though LMMS failed to improve the survival rate of mice from ovarian cancer, tissue based results in vibrated mice were suggestive for lower tumor incidence that involved fewer organ systems [46], suggesting that LMMS may interfere with the initiation but not the progression of cancer. In order to improve the preventive and/or inhibitory potential of LMMS, perhaps the signal is required to be optimized for bouts, amplitude and frequency based on tissue and/or cancer type.

Our results suggested that mechanical loads have a direct effect on the growth of cancer cells however the hypothesis that mechanical regulation on cancer cells would be obstructed by the physical compliance of these cells requires further testing. Increased compliance is a hallmark of cancer types of various tissues including breast [47], bladder [48], leukemia [49], ovarian [50], gastrointestinal [51] and lung [52] cancers. Cancer cells that show the highest potential for invasion are the cells with the highest compliance [27]. Metastatic capacity is also inversely related to the adhesion strength as metastatic cells lose adhesive properties to the extracellular matrix [53]. Altered physical properties of whole cancer cells therefore should be permissive towards under- or un-loading from the physical loads that are omnipresent in tissues. Consistent with the hypothesis that cancer cells are exposed to reduced mechanical loads, highly metastatic cells were shown to have reduced force interactions with each other and their environment [54]. As mechanical forces influence numerous functions in cells [55], the ability of cancer cells to mitigate this influence may affect their survival, proliferation and invasion.

Though we hypothesized a direct link between mechanical loads and cancer cell proliferation, other factors need to be addressed that may compliment or solely dominate the relationship between exercise and cancer. For example, exercise decreases adipose mass, a tissue that can store carcinogens [56–59] and therefore increase the risk for cancer. Further, fat mass is a determinant of fertility, which presents another risk factor for cancer in females [60]. Exercise also lowers circulating levels of insulin, which may act as a growth factor that enhance cell proliferation and inhibit cell death [61,62]. Lastly, exercise augments immune function [63,64], which may in turn increase an organism’s capability to determine and kill cancerous cells. Direct involvement of mechanical loads in determining the fate of cancer cells may act in tandem with the anti-carcinogenic repertoire that mechanical loads can foster.

## Conclusion

This study serves as a proof-of-principle that regular application of brief daily mechanical stimulus negatively affects the uncontrolled growth of aggressive type breast cancer cells *in vitro*. Although the mechanisms pertaining to this interaction remains largely elusive, follow up studies warrant further attention to regulation of molecular mechanisms in cancer cells by mechanical loads. If indeed mechanical loading of cancer cells by oscillatory motions is effective in reducing the growth of cancer, this non-invasive approach may be utilized alone or complementary with other therapies to combat against different types of cancer in clinic.

## Abbreviations

LMMS: Low magnitude mechanical stimulus
FBS: Fetal bovine serum
g: gravity
MTT: (3-(4,5-dimethylthiazol-2-yl)-2,5-diphenyltetrazolium bromide
DMEM: Dulbecco’s modified eagle medium
PI: Propidium iodide
DMSO: Dimethyl sulfoxide
PBS: Phosphate-buffered saline
EtOH: Ethanol
